# Comparative evaluation of prophylactic strategies for postpartum hemorrhage in vaginal delivery

**DOI:** 10.1002/ijgo.70636

**Published:** 2025-11-04

**Authors:** Stefania Fieni, Giovanni Morganelli, Alissa Valenti, Debora Formisano, Gabriella Maria Celora, Biancamaria Mastrandrea, Tullio Ghi

**Affiliations:** ^1^ Department of Obstetrics and Gynecology University of Parma Parma Italy; ^2^ Azienda Unità Sanitaria Locale—IRCCS Tecnologie Avanzate e Modelli Assistenziali in Oncologia di Reggio Emilia Reggio Emilia Emilia‐Romagna Italy; ^3^ Department of Women, Child and Public Health, Catholic University of Sacred Heart Rome Fondazione Policlinico Universitario Agostino Gemelli IRCCS Rome Italy

**Keywords:** oxytocin, postpartum hemorrhage, prevention, third stage of labor, vaginal delivery

## Abstract

**Objective:**

To compare the efficacy of different prophylactic oxytocin regimens in preventing postpartum hemorrhage (PPH) after vaginal delivery.

**Methods:**

Single‐center retrospective cohort study including all vaginal deliveries between February 1, 2022, and December 31, 2023, at a tertiary referral unit. Throughout the study period, the local protocol for PPH prevention in vaginal delivery was changed from oxytocin 10 IU intramuscular injection (IM) to 5 IU intravenous bolus (IV) and eventually to 10 IU IV. Data regarding base maternal characteristics, pregnancy course, labor, and maternal outcomes were retrospectively collected from institutional labor ward registries. The incidence of PPH was compared among the three historical cohorts who received different oxytocin regimens (10 IU IM, group A; 5 IU IV, group B; and 10 IU IV, group C) following propensity score matching for those variables that proved to be significantly associated with PPH.

**Results:**

During the study period, 3850 women had a vaginal birth at our tertiary care unit (1245 in the 10 IU IM group, 1291 in the 5 IU IV group and 1314 in the 10 IU IV group) and were enrolled in the study population. Of these, 688 (17.8%) had PPH. At multivariable logistic regression nulliparity, second‐degree or higher perineal tears, episiotomy, manual placental removal, birth weight, and multiple gestation appeared to be independently associated with PPH. PPH incidence was then compared among the groups following 1:1 propensity score matching for the above cited factors and appeared significantly higher with the use of 10 IU IM (group A) versus 5 IU IV (group B) oxytocin (21.3% versus 11.0%; *P* < 0.001) and versus 10 IU IV (group C) (22.1% versus 16.8%; *P* = 0.033); non‐significant differences between the incidence of PPH were observed when comparing the two regimens of IV administration.

**Conclusion:**

Intravenous administration of 5 IU or 10 IU is more effective than IM administration of 10 IU in reducing the incidence of PPH after vaginal deliveries. The efficacy of the two IV regimens appears similar.

## 1 INTRODUCTION

Postpartum hemorrhage (PPH) is an obstetric emergency and one of the leading causes of maternal mortality in both high‐ and low‐resource settings, although the absolute risk of death from PPH is significantly lower in the former group (from 0.001% to 0.01%).^1^ Active management of the third stage of labor is recommended to prevent this complication and is mostly based on the prophylactic administration of oxytocin at shoulder delivery.^2^

Recent research has investigated the efficacy of different routes of oxytocin administration in preventing PPH following vaginal delivery, reporting higher effectiveness for intravenous (IV) administration when compared with the intramuscular (IM) route.^3^ However, no data are available on the minimum effective dose.

According to the World Health Organization (WHO), oxytocin 10 IU (either IV or IM) is recommended following vaginal delivery for the prevention of PPH.^4^

This study aimed to compare the historically used regimen of 10 IU IM oxytocin with IV administration of 5 and 10 IU oxytocin to determine the most effective route of administration and the minimum effective dose to prevent PPH following vaginal birth.

## 2 MATERIALS AND METHODS

This retrospective observational study was conducted at the Maternity Hospital of the University of Parma, Italy, following approval from the local Ethical Committee (code protocol 153/2025/OSS/AOUPR—REMOVE24). Considering the retrospective and observational design of the study, a specific informed consent form was not collected because of the large size of the study sample, in accordance with the applicable regulations. The study was conducted on routinely collected data for clinical audit purposes, after local Ethical Committee approval, authorization and publication of the Data Protection Impact Assessment.

### 2.1 Study population and patient selection

We included all women who gave birth vaginally between February 1, 2022, and December 31, 2023, and received active management of the third stage of labor with oxytocin administration. Patients submitted to elective or emergency cesarean sections and those not eligible for oxytocin administration because of intolerance were not included.

During the study period, the institutional protocols for active management of the third stage of labor were periodically updated. Historically, 10 IU IM oxytocin was administered at shoulder delivery, as per the Italian national guidelines on intrapartum care.^5^ From October 1, 2022, the prophylactic oxytocin regimen was switched to an IV bolus of 5 IU as recommended by updated guidelines on prevention and treatment of PPH in cesarean section by the Italian Institute of Health, the Royal College of Obstetricians and Gynecologists, and the National Institute for Health and Care Excellence.^5^, ^6^, ^7^ Subsequently, from June 1, 2023, the dose was increased to a 10 IU IV bolus, in accordance with the American College of Obstetricians and Gynecologists guidelines for PPH prophylaxis following vaginal delivery.^8^

Active management of the third stage of labor, including uterotonics administration, early mother‐baby skin‐to‐skin contact (when possible), delayed cord clamping, and controlled cord traction,^9^ was consistently performed in all the groups. Blood loss was systematically estimated using graduated bags and gauze weighing. PPH was classified as minor, major, or massive depending on the amount of estimated blood loss (500–999, 1000–1499, and ≥1500 mL, respectively).

Clinical and epidemiologic data for each patient were collected from delivery ward records, including data regarding maternal characteristics (age, pre‐pregnancy body mass index [calculated as weight in kilograms divided by the square of height in meters], ethnicity, parity), pregnancy course (number of fetuses, gestational age at delivery, induction of labor), labor (epidural analgesia, labor augmentation, labor duration, mode of delivery, placental delivery method, postpartum blood loss, birth weight), oxytocin administration (route and dose), perineal outcomes (episiotomy and/or vaginal‐perineal lacerations).

The primary aim of the current study was to compare the incidence of PPH (blood loss >500 mL) among the three historical groups who received a different oxytocin regimen: 10 IU IM (group A), 5 IU IV (group B), and 10 IU IV (group C).

Propensity score matching was used to balance confounders between groups, ensuring a near‐randomized effect. Matching was performed with a 1:1 ratio using a caliper of 0.1.

### 2.2 Statistical analysis

Data were digitized into an anonymized spreadsheet using a unique numeric code. Risk factors for PPH were analyzed within each cohort using univariate analysis. Continuous variables were compared using Student *t*‐test or Mann–Whitney *U*‐test as appropriate, and categorical variables were analyzed using the χ^2^ test or Fisher exact test, as appropriate. Those variables that were statistically significant at univariate analysis were subsequently analyzed in a multivariable logistic regression model to test the impact of each factor on the risk of PPH. Post‐match univariate analysis was performed to evaluate differences in PPH incidence between the matched cohorts.

SPSS Statistics, Version 21.0 or later (IBM, Armonk, NY, USA), was used for data analysis.

## 3 RESULTS

**FIGURE 1:**
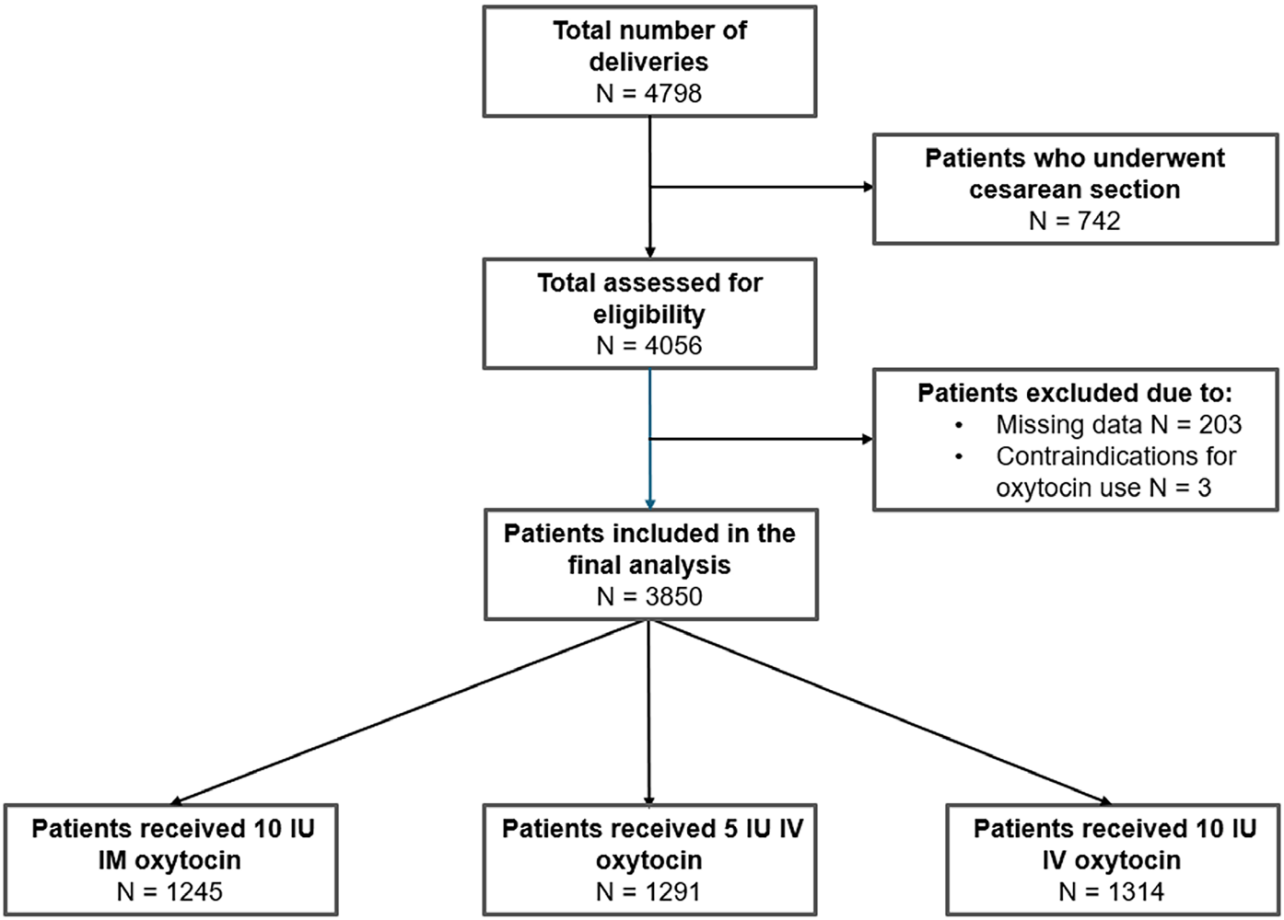
Flowchart illustrating the study population and cohort distribution.

During the study period, a total of 4798 women gave birth at our Maternity Unit. After excluding 742 women who underwent cesarean section, 4056 vaginal deliveries were analyzed. Of these, 203 (5.0%) were excluded because of incomplete data, and three were excluded because of contraindications to oxytocin administration (Figure 1).

The final sample consisted of 3850 patients, split into the following groups: group A, 1245 patients administered 10 IU IM oxytocin; group B, 1291 patients administered 5 IU IV oxytocin; and group C, 1314 patients administered 10 IU IV oxytocin.

Initially, to evaluate which factors were significantly associated with the occurrence of PPH, pairwise comparisons were conducted in the overall population between patients with and without a blood loss greater than 500 mL (Table 1). Patients who experienced PPH exhibited significantly higher gestational age at delivery (39.7 ± 1.67 versus 39.5 ± 1.72 weeks; *P* = 0.009); longer first stage (240 versus 180 min; *P* < 0.001) and second stage (62 versus 31 min; *P* < 0.001) of labor; increased rates of multiple gestation (1.5% versus 0.5%; *P* < 0.001), nulliparity (63.2% versus 48.8%; *P* < 0.001), labor induction (45.3% versus 34.9%; *P* < 0.001), epidural analgesia (58.4% versus 45.3%; *P* < 0.001), operative vaginal delivery (10.6% versus 5.9%; *P* < 0.001), second‐degree or higher perineal lacerations/episiotomy (48.4% versus 27.9%; *P* < 0.001), and manual placental removal (13.1% versus 7.2%; *P* < 0.001); and higher birth weight (3464 ± 514 versus 3302 ± 487 g; *P* < 0.001).

**TABLE 1:** Univariate analysis of factors associated with postpartum hemorrhage in the total cohort.^a^

Variables	Physiologic (*n* = 3162)	PPH (*n* = 688)	*P* value
Age, year	32.0 ± 5.22	31.9 ± 5.48	0.793
BMI	23.9 ± 4.97	24.1 ± 4.74	0.548
Gestational age at delivery, week	39.5 ± 1.72	39.7 ± 1.67	0.003
Multiple gestation	17 (0.5)	10 (1.5)	0.009
Nulliparity	1542 (48.8)	435 (63.2)	<0.001
Induction of labor	1105 (34.9)	312 (45.3)	<0.001
Length of first stage, min	180 (95–315)	240 (135–400)	<0.001
Length of second stage, min	31 (13–85)	62 (20–147)	<0.001
Epidural analgesia	1431 (45.3)	402 (58.4)	<0.001
Mode of delivery			
Vaginal delivery	2975 (94.1)	615 (89.4)	<0.001
Assisted vaginal delivery	187 (5.9)	73 (10.6)	
Perineal tears			
None/first‐degree	2279 (72.1)	355 (51.6)	<0.001
Episiotomy/second‐degree or higher	883 (27.9)	333 (48.4)	
Manual placenta removal	227 (7.2)	90 (13.1)	<0.001
Birth weight, g	3302 ± 487	3464 ± 514	<0.001

Abbreviations: BMI, body mass index (calculated as weight in kilograms divided by the square of height in meters); PPH, postpartum hemorrhage.

^a^ Data are presented as mean ± standard deviation, median (interquartile range), or number (percentage).

Binomial logistic regression identified nulliparity, severe perineal lacerations/episiotomy, manual placental removal, and birth weight as independent risk factors for PPH (Table 2).

**TABLE 2:** Binomial logistic regression of factors associated with postpartum hemorrhage in the total cohort.

Variables	aOR	95% CI	*P* value
Age	1.01	0.986–1.04	0.356
BMI	1.01	0.984–1.04	0.408
Gestational age at delivery	0.92	0.816–1.03	0.128
Multiple gestation	1.20	0.036–1.23	0.083
Nulliparity	1.80	1.298–2.50	<0.001
Induction of labor	1.17	0.878–1.56	0.285
Length of first stage	1.00	0.999–1.000	0.298
Length of second stage	1.00	0.999–1.000	0.226
Epidural analgesia	1.18	0.862–1.61	0.302
Assisted vaginal delivery	1.20	0.73–1.93	0.464
Episiotomy/second‐degree or higher tears	2.16	1.623–2.86	<0.001
Manual placenta removal	2.38	1.598–3.53	<0.001
Birth weight	1.90	1.001–2.000	<0.001

Abbreviations: aOR, adjusted odds ratio; BMI, body mass index (calculated as weight in kilograms divided by the square of height in meters); CI, confidence interval.

Patients from group A (10 IU IM), group B (5 IU IV), and group C (10 IU IV) were matched in a 1:1 ratio for the variables independently associated with the occurrence of PPH after logistic regression analysis and compared.

A significantly lower incidence of PPH was found between patients receiving 5 IU IV (group B) versus 10 IU IM (group A) oxytocin (11% versus 21.3%; *P* < 0.001) and between those receiving 10 IU IV (group C) versus 10 IU IM (group A) oxytocin (16.8% versus 22.1%; *P* = 0.033) whereas no significant difference in the incidence of PPH was observed between patients receiving 5 IU IV or 10 IU IV oxytocin (10.9% versus 13.8%; *P* = 0.172) (Table 3).

**TABLE 3:** Pairwise comparisons between patients administered 10 IU IM versus 5 IU IV bolus (Group A vs. Group B), patients administered 10 IU IM versus 10 IU IV bolus (Group A vs. Group C), and patients administered 5 IU IV versus 10 IU IV bolus (Group B vs. Group C) after propensity score matching.^a^

Variables	Group A vs. Group B			Group A vs. Group C			Group B vs. Group C		
	10 IU IM (*n* = 464)	5 IU IV (*n* = 464)	*P* value	10 IU IM (*n* = 512)	10 IU IV (*n* = 512)	*P* value	5 IU IV (*n* = 485)	10 IU IV (*n* = 485)	*P* value
Age, year	32.1 ± 5.43	31.9 ± 5.06	0.688	32.2 ± 5.38	31.7 ± 5.33	0.177	31.8 ± 5.10	31.7 ± 5.32	0.753
BMI	23.8 ± 4.47	23.4 ± 4.61	0.308	23.9 ± 4.98	24.0 ± 5.14	0.773	24.0 ± 5.13	24.4 ± 4.79	0.428
Gestational age at delivery, week	39.8 ± 1.24	39.8 ± 1.13	0.877	39.7 ± 1.31	39.8 ± 1.18	0.193	39.8 ± 1.20	39.7 ± 1.17	0.624
Multiple gestation	0	2 (0.4)	0.157	0	0	‐	1 (0.2)	1 (0.2)	1.000
Nulliparity	219 (52.8)	219 (52.8)	1.000	273 (53.3)	273 (53.3)	1.000	244 (50.3)	244 (50.3)	1.000
Induction of labor	147 (31.7)	165 (35.6)	0.211	158 (30.9)	173 (33.8)	0.316	165 (34.0)	178 (36.7)	0.383
Length of first stage, min	188 (100–380)	190 (98–375)	0.840	187 (90–380)	179 (90–285)	0.164	185 (105–360)	160 (90–268)	0.113
Length of second stage, min	34 (15–107)	33 (13–85)	0.434	39 (15–97)	39 (11–100)	0.665	27 (12–80)	31 (13–97)	0.557
Epidural analgesia	176 (38.0)	212 (46.0)	0.014	220 (43.1)	258 (50.5)	0.017	226 (46.8)	242 (50.1)	0.303
Mode of delivery			0.762			0.091			0.195
Vaginal delivery	442 (95.3)	440 (94.8)		487 (95.1)	474 (92.6)		464 (95.7)	455 (93.8)	
Assisted vaginal delivery	22 (4.7)	24 (5.2)		25 (4.9)	38 (7.4)		21 (4.3)	30 (6.2)	
Perineal tears			1.000			1.000			1.000
None/first‐degree	340 (73.3)	340 (94.8)		383 (74.8)	383 (74.8)		375 (77.3)	375 (77.3)	
Episiotomy/second‐degree or higher	124 (26.7)	124 (26.7)		129 (25.2)	129 (25.2)		110 (22.7)	110 (22.7)	
Manual placenta removal	7 (1.5)	7 (1.5)	1.000	1 (0.2)	1 (0.2)	1.000	10 (2.1)	10 (2.1)	1.000
Birth weight, g	33 704 ± 359	3374 ± 359	1.000	3359 ± 350	3359 ± 350	1.000	3358 ± 364	3358 ± 364	1.000
PPH	99 (21.3)	51 (11.0)	<0.001	113 (22.1)	86 (16.8)	0.033	53 (10.9)	67 (13.8)	0.172
Minor	83 (83.8)	38 (74.5)	0.438	98 (86.7)	65 (75.6)	0.100	39 (73.6)	48 (71.6)	0.976
Major (1000–1500 mL)	13 (13.1)	10 (19.6)		9 (8.0)	15 (17.4)		8 (15.1)	12 (17.9)	
Massive (>1500 mL)	3 (3.0)	3 (5.9)		6 (5.3)	6 (7.0)		6 (11.3)	7 (10.5)	

Abbreviations: BMI, body mass index (calculated as weight in kilograms divided by the square of height in meters); IM, intramuscularly; IV, intravenously; PPH, postpartum hemorrhage.

^a^ Data are presented as mean ± standard deviation, median (interquartile range), or number (percentage).

Additionally, compared with group A (10 IU IM) the administration of epidural analgesia was significantly more frequent in group B (5 IU IV) (46% versus 38%; *P* = 0.014) and group C (10 IU IV) (50.5% versus 43.1%; *P* = 0.017).

No significant differences in the grade of PPH (minor, major, massive) were found among the groups.

## 4 DISCUSSION

This retrospective, observational study, conducted on historical cohorts of patients matched by propensity score analysis, showed that the IV administration of oxytocin as a bolus of either 5 or 10 IU after vaginal birth was more effective in reducing the incidence of PPH when compared with a 10 IU IM injection. Notably, the IV administration of 10 IU compared with 5 IU was not found to be superior in preventing a blood loss greater than 500 mL.

The efficacy of IV oxytocin administration for PPH prevention in patients undergoing cesarean section has been largely investigated, but few randomized studies on the efficacy of PPH prophylaxis following vaginal delivery have been conducted.^3^, ^9^, ^10^, ^11^ Available evidence suggests greater effectiveness for the IV route compared with the IM route, and this aligns with the findings of our study.

A meta‐analysis of seven randomized studies, including 7817 participants and comparing the efficacy of IV and IM oxytocin administration in reducing blood loss during the third stage of labor, reported that IV administration is associated with a significantly lower risk of PPH (5.6% versus 7.2%, relative risk [RR] 0.78, 95% confidence interval [CI] 0.66–0.92) and need for blood transfusions (0.6% versus 1.3%, RR 0.44, 95% CI 0.26–0.77). Furthermore, a reduction in severe maternal morbidity (0.3% versus 0.6%, RR 0.47, 95% CI 0.22–1.00) and PPH of 1000 mL or greater (1.5% versus 2.3%, RR 0.65, 95% CI 0.39–1.08) were noted, whereas adverse effects profiles were similar between the groups.^3^

With regard to dosages, previous research has compared the efficacy among various IV regimens following cesarean delivery; however, to our knowledge, only one study has been conducted after vaginal birth. A double‐masked randomized trial comparing three different prophylactic dosages of oxytocin (80, 40, or 10 IU in 500 mL saline infused over 1 h) including almost 1800 vaginal deliveries, found no significant differences in the incidence of PPH requiring treatment (administration of therapeutic uterotonics, blood transfusion, uterine tamponade, embolization, or surgery), which occurred in 6%–7% of the cases in each group.^11^

In a sub‐analysis on 2901 patients included in their meta‐analysis, Salati et al.^12^ found that the administration of 10 IU IV oxytocin reduced the need for supplementary drugs when compared with lower dosages (RR 0.48, 95% CI 0.33–0.68). However, most of the studies included in this review were not blinded; hence, high risk of bias was reported by the authors themselves. Furthermore, the cumulative study population given doses less than 10 IU was smaller when compared with the population given 10 IU (273 versus 2901 patients); this aspect raises concerns on the generalizability of the reported findings.^12^

The present study is the first study reporting non‐significant differences in the incidence of PPH when comparing a 5 IU with a 10 IU IV bolus after vaginal delivery.

After propensity score matching, a statistically significant difference in the rate of patients undergoing epidural analgesia was reported when comparing both groups B and C (5 and 10 IU IV) with group A (10 IU IM), respectively. This finding may be the result of increased access to epidural analgesia at our center during the second period of the study. However, epidural analgesia was not independently associated with PPH when investigating risk factors in the multivariable analysis; hence, it is unlikely that it might have influenced the incidence of PPH.

Results from the present study confirm that IV oxytocin administration is more effective in preventing PPH after vaginal delivery when compared with IM injection. Previous studies reported that IM administered oxytocin starts its uterotonic effect 3–7 min after administration; in contrast, IV administration has been proven to have immediate effect on uterine tone.^13^

On this basis, it seems appropriate to replace the IM oxytocin injection with IV administration as a standard of care to enhance PPH prevention after vaginal delivery.

Our study looked at the incidence of PPH as the principal outcome of interest, but based on our findings, dosages of IV oxytocin greater than 5 IU seem to bring no significant improvement in the rate of PPH after vaginal delivery.

This is in contrast to the most recent WHO guidelines, which for the IV route recommend the administration of 10 IU of oxytocin but do not provide clear evidence to support this.^4^

Establishing the minimum effective dose of IV oxytocin to prevent PPH after vaginal delivery is a key factor as the adverse effects from IV oxytocin administration are likely be dose dependent and are known to clinicians.

Among these, hypotension, arrhythmia, and other cardiovascular events have been reported to potentially occur in patients without pre‐existing cardiovascular risk factors.^14^, ^15^ A slow injection over 3 min is recommended to mitigate cardiovascular responses (e.g. reduction in blood pressure and systemic vascular resistance index, increased cardiac and left ventricular workload indices) associated with IV bolus administration.^16^

Our study makes a contribution to the field and suggests that in the general population 5 IU of oxytocin administered IV may be equally effective to 10 IU in preventing PPH at vaginal delivery.

Regarding the strengths of the present study, the collection of large cohorts in a relatively short time is likely to minimize the possibility that changes in local practice could have influenced the outcome. Furthermore, propensity scoring analysis and matching helped to control the effect of confounding factors on the incidence of PPH observed across the groups.

The major limitation of our study is its retrospective design. Additionally, as a result of the incompleteness of data, our analysis has not included other outcomes of clinical interest (e.g. adverse effects associated with IV oxytocin infusion compared with the IM route, post‐delivery hemoglobin levels, need for blood transfusions).

In conclusion, the present study shows that IV oxytocin is more effective than IM oxytocin in preventing the occurrence of PPH following vaginal delivery. Moreover, IV boluses of 5 or 10 IU seem to have comparable efficacies. Further well‐designed studies are needed to obtain higher quality evidence, which may lead to a more appropriate management of the third stage of labor.

## AUTHOR CONTRIBUTIONS

SF, GM, and TG contributed to conceptualization; GM, AV, GMC, and BM contributed to data curation; SF and TG supervised the study; SF, GM, DF, and TG contributed to the methodology; GM and DF performed the formal analysis; all the authors contributed to the investigation; SF and AV wrote the original draft; and GM, GMC, BM, and TG performed the reviewing and editing. All authors have read and agreed to the final version of the manuscript.

## CONFLICT OF INTEREST STATEMENT

The authors have no conflicts of interest.

## Data Availability

The data that support the findings of this study are available on request from the corresponding author. The data are not publicly available because of privacy or ethical restrictions.
